# Identification of a Ruminant Origin Group B Rotavirus Associated with Diarrhea Outbreaks in Foals

**DOI:** 10.3390/v13071330

**Published:** 2021-07-09

**Authors:** Tirth Uprety, Chithra C. Sreenivasan, Ben M. Hause, Ganwu Li, Solomon O. Odemuyiwa, Stephan Locke, Jocelynn Morgan, Li Zeng, William F. Gilsenan, Nathan Slovis, Laurie Metcalfe, Craig N. Carter, Peter Timoney, David Horohov, Dan Wang, Erdal Erol, Emma Adam, Feng Li

**Affiliations:** 1 Maxwell H. Gluck Equine Research Center, University of Kentucky, Lexington, KY 40546, USA; TUP226@uky.edu (T.U.); chithra.sreenivasan@uky.edu (C.C.S.); ptimoney@uky.edu (P.T.); dwhoro2@uky.edu (D.H.); dan.wang@uky.edu (D.W.); 2 Department of Veterinary and Biomedical Sciences, South Dakota State University, Brookings, SD 57007, USA; benjamin.hause@sdstate.edu; 3 Department of Veterinary Diagnostic and Production Animal Medicine, College of Veterinary Medicine, Iowa State University, Ames, IA 50011, USA; liganwu@iastate.edu; 4 Veterinary Medical Diagnostic Laboratory, College of Veterinary Medicine, University of Missouri, Columbia, MO 65212, USA; odemuyiwas@missouri.edu; 5 Veterinary Diagnostic Laboratory, University of Kentucky, Lexington, KY 40512, USA; slocke@uky.edu (S.L.); jocelynn.morgan@uky.edu (J.M.); li.zeng@uky.edu (L.Z.); craig.carter@uky.edu (C.N.C.); 6 Rood and Riddle Equine Hospital, Lexington, KY 40511, USA; bgilsenan@roodandriddle.com (W.F.G.); lmetcalfe@roodandriddle.com (L.M.); 7 Hagyard Equine Medical Institute, Lexington, KY 40511, USA; nmslovis@yahoo.com

**Keywords:** rotavirus B, foal, diarrhea, outbreak

## Abstract

Equine rotavirus group A (ERVA) is one of the most common causes of foal diarrhea. Starting in February 2021, there was an increase in the frequency of severe watery to hemorrhagic diarrhea cases in neonatal foals in Central Kentucky. Diagnostic investigation of fecal samples failed to detect evidence of diarrhea-causing pathogens including ERVA. Based on *Illumina*-based metagenomic sequencing, we identified a novel equine rotavirus group B (ERVB) in fecal specimens from the affected foals in the absence of any other known enteric pathogens. Interestingly, the protein sequence of all 11 segments had greater than 96% identity with group B rotaviruses previously found in ruminants. Furthermore, phylogenetic analysis demonstrated clustering of the ERVB with group B rotaviruses of caprine and bovine strains from the USA. Subsequent analysis of 33 foal diarrheic samples by RT-qPCR identified 23 rotavirus B-positive cases (69.69%). These observations suggest that the ERVB originated from ruminants and was associated with outbreaks of neonatal foal diarrhea in the 2021 foaling season in Kentucky. Emergence of the ruminant-like group B rotavirus in foals clearly warrants further investigation due to the significant impact of the disease in neonatal foals and its economic impact on the equine industry.

## 1. Introduction

Rotavirus (RV) is a common enteric pathogen associated with diarrhea in newborn children, animals, and birds [1,2]. Globally, rotavirus is the leading cause of diarrhea in children less than 5 years old [3]. The virus is a non-enveloped, double-stranded RNA virus that belongs to the Reoviridae family [4]. The RV genome comprises 11 dsRNA segments, with each segment dedicated to coding for one protein (segment 11 is an exception; it codes for two non-structural proteins: NSP5 and NSP6). In most rotaviruses, segments 1, 2, 3, 4, 6, and 9 code for VP1, VP2, VP3, VP4, VP6, and VP7, respectively, while segments 5, 8, 7, 10, and 11 code for non-structural proteins NSP1–NSP6, respectively [5]. During viral replication, proteolytic enzymes such as trypsin cleave VP4 into VP5^*^ and VP8^*^, which drives viral maturation and allows the virus to spread among exposed host species. The numbering of genome segments is based on the migration pattern of RNA segments in analytical gels that can vary between strains [6].

The RV virion is an icosahedral, triple-layered particle (TLP). The innermost capsid layer is formed by VP2 protein, while VP6 forms the middle capsid layer. VP7 forms the outermost capsid layer through which the spike protein VP4 protrudes [7]. VP6 is most conserved among different strains within a RV group [8]. As a result, its sequence-based demarcation has been used extensively for RV group classification [9]. Based on the VP6 sequence and antigenic differences, the RV genus is divided into species (also called groups/serogroups); currently there are nine groups, namely A, B, C, D, F, G, H, I, and J [10]. The outer capsid protein VP7 and spike protein VP4 are the major viral antigens that elicit neutralizing antibody responses, and which form the basis of the dual classification system for rotavirus group A (RVA). Based on serological assays and nucleotide sequences of outer capsid protein VP7 and spike protein VP4, RVA is further classified into G and P serotypes [11]. So far, 36 G serotypes and 51 P genotypes have been reported (Rotavirus Classification Working Group).

Although RVA is the most important causative agent associated with gastroenteritis and severe diarrhea in both children and newborn animals, rotavirus groups B (RVB), C (RVC), and H (RVH) can also cause clinical diarrhea in both humans and animals [2,12,13,14]. Rotavirus groups D (RVD), F (RVF), and G (RVG) have been associated mainly with avian disease [15]. Interestingly, unlike RVA, RVB has the ability to cause outbreaks of a “cholera-like diarrhea” in adults, as well as infants [16,17,18]. Sporadic or epidemic outbreaks of diarrhea associated with RVB were also reported in calves, adult cows, lambs, kids and adult goats, and piglets [19]. Zoonotic transmission of RVA has been well established with frequent reports of interspecies transmission [20,21]. The segmented nature of its genome allows for both genomic reassortment and recombination, which can lead to the emergence of new antigenic variants [22,23]. Increasing evidence indicates reassortment events are not constrained among animal strains and that reassortment between animals and human strains can occur [24,25,26,27]. It is generally believed that RVA reassortants can jump species and infect and spread in new host species. Thus, continuous surveillance and monitoring of RVs in both animals and humans are essential for the prevention of future diarrhea outbreaks as well as for the implementation of timely countermeasure strategies. 

Equine rotavirus group A (ERVA) is one of the most common causes of severe dehydrating diarrhea in foals less than 3 months old, and its prevalence can vary from 20% to as high as 77% in outbreaks of the disease [28]. To date, six G types (G3, G14, G5, G8, G10, G13) and six P types (P(1), P(3), P(7), P(11), P(12), P(18)) have been reported to be associated with outbreaks of clinical diarrhea in foals [29,30,31,32,33,34,35,36]. Of these genotypes, G3P(12) and G14P(12) are the most prevalent genotypes around the world. Equine G3P(12) genotypes were isolated in 1975, whereas equine G14P(12) genotypes were discovered in 2005 [28,37,38]. In addition to posing a significant health concern to the equine industry, ERVA, especially those G3 genotype-derived reassortants, has been found to spread and cause clinical diarrhea in children [39]. Cross-species transmission has also been described for infrequently occurring equine rotavirus A strains like G5P(7) and G3P(3) involving spillover events from pigs and cats to horses, respectively [40,41,42]. Intriguingly, despite frequent detection of RVB in human diarrhea cases as well as in a variety of agricultural animal species, including calves and goats, research on RVB and the associated diarrhea in horses is very limited. To date, only a single study reported on the presence of RVB in horses in Germany [43]. This early work with a focus on the detection of rotavirus species A, B, and C in domestic animals found that one out of a total of 37 equine samples (2.7% detection rate) tested positive for RVB in an RT-PCR assay. The associated disease status in that RVB-positive horse and viral genome sequence were not investigated in that study.

In this study, we investigated the causative agent(s) of a series of diarrhea outbreaks that occurred in the 2021 foaling season in Kentucky, USA, in neonatal foals at approximately 24–72 h of age. Other clinical signs included inappetence, mild colic, and transient ileus. Affected foals were largely born to mares that received prior immunization with a commercial inactivated equine rotavirus group A G3P(12) vaccine (Zoetis). ERVA and other significant diarrhea-causing pathogens were negative in most cases of diarrhea in the course of this investigation. Using next-generation sequencing, we identified a novel group B rotavirus in feces and fecal swabs from foals suffering from watery diarrhea. Furthermore, we determined the complete genome sequence of this ERVB from pooled fecal/swab samples, except for small portions of non-coding sequences at the 5′ and 3′ ends. Interestingly, the genomic analysis demonstrated that the novel virus exhibited more than 96% overall amino acid identity to ruminant RVB, indicating the possibility that the virus was ruminant in origin. These findings confirm for the first time the circulation of a group B rotavirus in horses in which it can be associated with enteric disease.

## 2. Materials and Methods

### 2.1. Ethics Statement

Foal feces or fecal swab samples were collected as part of a routine diagnostic investigation by licensed veterinarians and submitted to the University of Kentucky’s Veterinary Diagnostic Laboratory or the Gluck Equine Research Center, Lexington, KY, USA.

### 2.2. Fecal Sample Collection and Viral Metagenomic Sequencing

Feces were collected from three neonatal foals (2–7 days of age) with severe watery to hemorrhagic diarrhea. Approximately 1 g of feces from each foal was pooled together and used for metagenomic sequencing. In addition, fecal swabs were collected from four neonatal foals between 2 and 6 days old that suffered from diarrhea, and a pooled sample from these four fecal swabs was also included in the metagenomic sequencing analysis. Note that these fecal samples were derived from five equine farms affected with foal diarrhea during the 2021 foaling season, which are located in proximity to Lexington, KY, USA.

The standard Illumina MiSeq-based metagenomic sequencing method was used for the identification of the causative agent likely responsible for the outbreaks of idiopathic foal diarrhea. Briefly, clarified feces and fecal swab pooled samples were treated with nucleases followed by nucleic acid isolation. Reverse transcription and second-strand synthesis were performed with barcoded random hexamers followed by amplification with barcode primers. Sequencing libraries for each of the pooled samples were constructed with a Nextera XT library preparation kit (Illumina) followed by sequencing on a MiSeq instrument. Approximately 0.9–1.2 million paired 151 base pair (bp) reads were generated per pooled sample. Contigs were assembled de novo using CLC Genomics and analyzed by BLASTX using the BLAST2Go plugin and the non-redundant protein sequence database in May 2020.

### 2.3. Genome Sequencing and Analysis

Contigs encoding proteins with homology to ruminant RVB were identified by BLASTP analysis. The genome sequence of segments 1–11 of RVB/Horse-wt/USA/KY/1518 was submitted to Genbank under accession nos. MZ327688–MZ327698, respectively. Phylogenetic analyses were performed using MEGA X software [44]. Evolutionary analyses were conducted using the maximum-likelihood algorithm, and tree topology was verified by performing 1000 bootstrap replicates.

### 2.4. Transmission Electron Microscopy

Fecal samples from two foals suffering from watery diarrhea were resuspended in distilled water (10% suspension) and lysed in a tissue-lyser with beads. Following low-speed centrifugation, the supernatant was passed through a 450 nm filter. A total of 190 µL of the filtrate was subjected to ultracentrifugation (199 × 10^3^ g for 2 h) using an Airfuge air-driven ultracentrifuge (Beckman-Coulter, Indianapolis, IN, USA). Pellets (20 µL) were resuspended and negatively stained with 1.3% phosphotungstic acid followed by examination on a JEOL JEM 1400 transmission electron microscope (JEOL USA Inc, Peabody, MI, USA).

### 2.5. Detection of Equine Rotavirus Group B Genome in Clinical Samples

Forty-two feces and fecal swab samples (*n* = 42), originating from 22 equine farms in Central Kentucky, were analyzed for ERVB using RT-qPCR. Nine of the samples were from healthy foals, and 33 were from foals with watery or hemorrhagic diarrhea. Most of these neonatal foals were 2–7 days of age. We deployed two standard RT-qPCR assays for the molecular detection of ERVB in clinical samples. RT-qPCR assay I targeted the VP7 gene of ERVB with one pair of primers (forward, nucleotide region 651–670; reverse, nucleotide region 739–756) and a Taqman probe (nucleotide region 672–696, labeled with FAM dye at the 5′ end and BHQ at the 3′ end). RT-qPCR assay II involved NSP2-targeting primers (forward, nucleotide region 655–674; reverse, nucleotide region 788–807); and a probe (nucleotide region 703–725). The detailed sequences of primers and probes will be provided upon request.

Viral RNAs were extracted from clinical samples by using the PureLink viral RNA/DNA isolation kit (Invitrogen) according to the manufacturer’s instructions. RT-PCR was performed using the applied biosystem TaqMan RNA-to-Ct one-step kit in the ViiA 7 Real-Time PCR instrument (Applied Biosystems) for Assay I and Path-ID™ Multiplex One-Step RT-PCR Kit for Assay II (Thermofisher, Waltham, MA, USA).

Thermal cycling conditions for Assay I and for Assay II, were as follow: initial reverse transcription, 48 °C for 15 min (for Assay I) and 45 °C for 10 min (for Assay II); PCR activation, 95 °C for 10 min, followed by 40 cycles of 15 s at 95 °C, 60 s at 60 °C.

## 3. Results

### 3.1. Foal Diarrhea Outbreak

**Figure 1:**
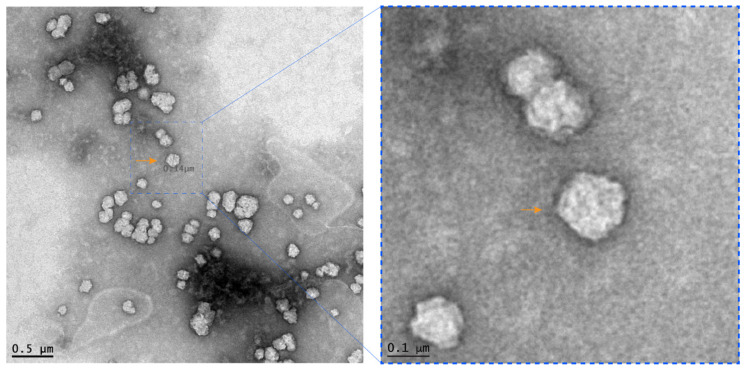
Transmission electron microscopy analysis of rotavirus particles in the feces of a diarrheic foal. Negative stain (the left image) shows single and clusters of round non-enveloped electron-lucent particles with electron-dense “surface holes” characteristic of rotavirus particles (Bar = 500 nm). Note that the right panel is the scaled-up image of the indicated field in the original TEM image (Bar = 100 nm).

Foal diarrhea cases were increasingly being reported in newborn foals aged 2–7 days from different farms in Central Kentucky, USA, at the beginning of the 2021 foaling season (February and March). Mares were vaccinated with an inactivated monovalent ERVA vaccine during their pregnancy according to the manufacturer’s recommendations (https://www.zoetisus.com, accessed on 16 June 2021). Foals developed diarrhea at approximately 48 h of age and diarrheic episodes typically lasted 3–4 days. Farms with a diarrhea problem experienced up to 100% morbidity, with each new foal born on the farm succumbing to disease following their index case suggesting a highly contagious nature of the disease. Farms able to break this pattern were those implementing strict biosecurity protocols. Foals required intensive medical care either on the farm or at referral hospitals in Lexington. The rapid and intense medical intervention provided by the veterinary care facilities in Central Kentucky enabled an extremely high survival rate in these cases. The clinical signs included inappetence, weakness, dehydration, severe electrolyte imbalance, and watery yellow and foul-smelling diarrhea. While hemorrhagic watery diarrhea was also noticed in some cases, there were no flecks of blood present in samples from affected foals. None of the dams of the foals developed diarrhea. Despite samples from foal diarrhea cases testing negative on an ERVA specific RT-qPCR assay, transmission electron microscope (TEM) examination of diarrhea samples from two diseased foals revealed single and clusters of round particles (~0.1 µm ø) with electron-dense “surface holes” characteristic of rotavirus particles (Figure 1). The combination of TEM and RT-qPCR data indicated that a non-A rotavirus was implicated in causing the series of neonatal foal diarrhea outbreaks.

Seven representative foal diarrhea samples collected from five equine farms in the Lexington, Kentucky area were tested using a common enteric pathogens foal and neonate GI/diarrhea panel (*Clostridium difficile* toxins A and B, equine coronavirus, *Lawsonia intracellularis*, *Salmonella* spp., *Cryptosporidium* spp., equine rotavirus group A, *Rhodococcus equi*, *Clostridium perfringens*). All seven samples tested negative for all of the agents included in the panel, except for three samples that were positive for *C. perfringens*. The detection of *Clostridium perfringens* in equine fecal samples may be insignificant, as these bacteria can also be found in samples from non-diarrheic healthy foals (data not shown).

### 3.2. Identification of a Novel Group B Rotavirus by Next-Generation Sequencing

The aforementioned seven samples negative on testing with the neonate GI/diarrhea panel, were further investigated by *Illumina* MiSeq-based deep RNA sequencing. Approximately 1 g of feces from each of three diarrheic foals was pooled together and used for the metagenomic sequencing. In addition, fecal swabs from the other four neonatal foals were pooled together in the same experiment. Sequencing libraries were constructed for each pool individually using a Nextera XT library preparation kit followed by sequencing on a MiSeq instrument using paired 150-bp reads. Sequenced reads were trimmed of adapter sequences using onboard software before being exported to CLC Genomics and assembled de novo. Contig sequences were analyzed by BLASTX using the BLAST2Go plugin incorporated into that software package.

For pooled feces, approximately 1,194,168 reads were generated, and 45.5% of the total sequence reads were mapped to ruminant RVB. Similarly, approximately 937,432 reads were generated for the fecal swab pool, and 72% of the total reads mapping to RVB. In-depth analysis of sequence reads failed to identify RVA or other viral or bacterial enteric pathogens of known concern as a cause of foal diarrhea. Total sequencing reads from each pool were used to assemble the respective full-length genome sequence of the ERVB, except for certain non-coding sequences at the termini of a few segments. We focused on fecal swab-derived consensus full-genome sequence for further analysis in consideration of more reads generated for the rotavirus B sequence and additional validation by separate metagenomic sequencing. The virus was provisionally designated RVB/Horse-wt/USA/KY/1518/2021.

As summarized in Table 1, nucleotide BLAST analysis of RVB/Horse-wt/USA/KY/1518/2021 revealed that segment 1 (VP1), segment 2 (VP2), segment 3 (VP3), segment 5 (NSP1), and segment 7 (NSP3) aligned best with segment 1, segment 2, segment 3, segment 5, and segment 7 of RVB/Goat-wt/USA/Minnesota-1/2016 with 93.19%, 97.82%, 95.04%, 95.75%, and 97.84% sequence identity, respectively. Similarly, segment 4 (VP4), segment 6 (VP6), segment 8 (NSP2), and segment 11 (NSP5/NSP6) of the equine virus showed 97.40%, 96.34%, 97.90%, and 96.41% sequence identity, respectively, with its corresponding segments in a different caprine virus, RVB/Goat-wt/USA/CA22/2014. It is intriguing that segment 9 (VP7) of RVB/Horse-wt/USA/KY/1518 scored best in sequence alignment with a bovine virus, RVB/Cow-wt/USA/MN10-1/2010 G3P[X], with 96.37% sequence identity. Furthermore, segment 10 (NSP4) of the equine virus had the most homology with its counterpart in another bovine virus, RVB/Cow-wt/JPN/IS-1/1999/G3P[X], with 94.26% sequence identity. In summary, the nucleotide BLAST analysis appears to indicate that the equine rotavirus B associated with the diarrhea outbreaks in foals may have evolved from ruminants.

De novo genome assembly and open reading frame (ORF) analysis found a single ORF for all 11 genome segments. Further protein sequence analysis showed that all segments had greater than 96% identity with ruminant group B rotaviruses represented by RVB/Goat-wt/USA/Minnesota-1/2016, RVB/Goat-wt/USA/CA22/2014, RVB/Cow/Nemuro, and RVB/Cow-wt/JPN/IS-2/2002/G3P[X] (Table 2). Segments with more than 99% homology to ruminant group B viruses were NSP3 (99.66%), VP7 (99.60%), NSP2 (99.33%), and VP2 (99.15%), while the most divergent segments were NSP1 (96.25%) and NSP4 (96.63%). Segments within 97–99% identity between equine and ruminant viruses included VP4 (97.10%), VP1 (97.15%), VP3 (97.38%), NSP5 (97.59%), and VP6 (98.98%). Comparative nucleotide and amino acid sequence analysis of all 11 segments supports the theory that the novel equine group B rotavirus originated from ruminants; this warrants further investigation.

### 3.3. Phylogenetic Analysis

**Figure 2:**
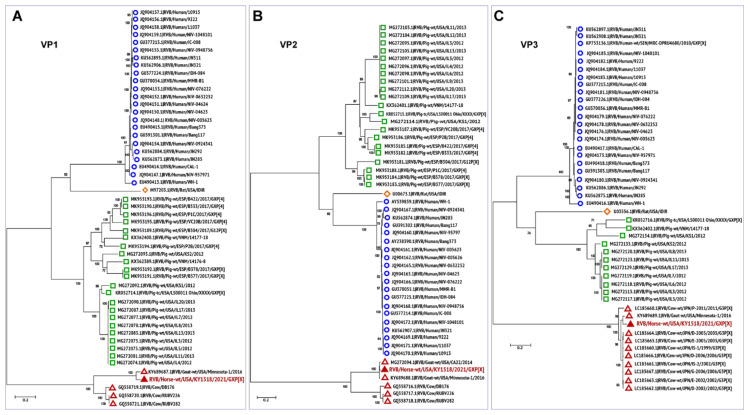
Phylogenetic trees of the complete protein-coding nucleotide sequences of the group B rotavirus VP4 (**A**), VP6 (**B**), and VP7 (**C**) genes. Maximum-likelihood analysis in combination with 1000 bootstrap replicates was used to derive trees based on the nucleotide sequences encoding respective proteins. The RVB strains are color-coded as human (open blue circle), murine (open orange diamond), porcine (open green square), and ruminant (open red triangle), with RVB/Horse-wt/USA/KY/1518/2021 (filled red triangle) highlighted in red font. A scale representing evolutionary distance that indicates the number of nucleotide substitutions per site is also shown in each panel. Bootstrap values are shown above branches to the left of major nodes.

**Figure 3:**
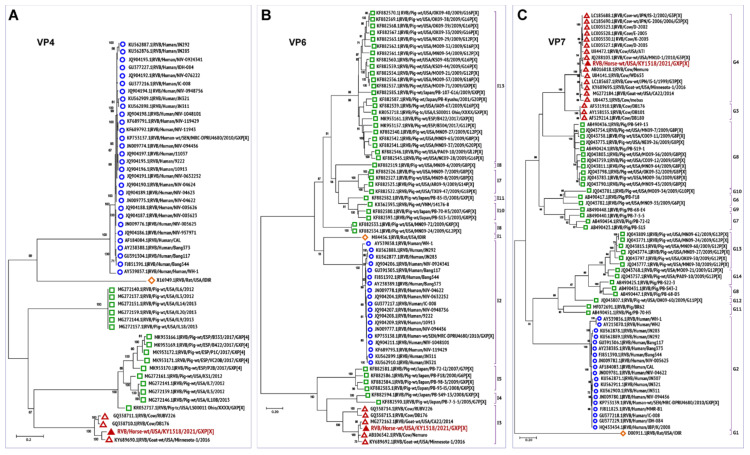
Phylogenetic trees of the complete protein-coding nucleotide sequences of the group B rotavirus VP4 (**A**), VP6 (**B**), and VP7 (**C**) genes. Maximum-likelihood analysis in combination with 1000 bootstrap replicates was used to derive trees based on the nucleotide sequences encoding respective proteins. The RVB strains are color-coded as human (open blue circle), murine (open orange diamond), porcine (open green square), and ruminant (open red triangle), with RVB/Horse-wt/USA/KY/1518/2021 (filled red triangle) highlighted in red font. The genotypes of RVB strains associated with VP6 and VP7 segments are also shown. The RVB VP6 (I) genotypes were based on the proposed 81% nucleotide and 89% amino acid identity cut-off values. The known VP7 (G) genotypes from G1–14 associated with RVB strains are also shown [27,45]. The distantly related porcine RVB strains of G15–23 genotypes were not included in the VP7 phylogenetic analyses. A scale representing evolutionary distance indicates the number of nucleotide substitutions per site is also shown in each panel. Bootstrap values are shown above branches to the left of major nodes.

**Figure 4:**
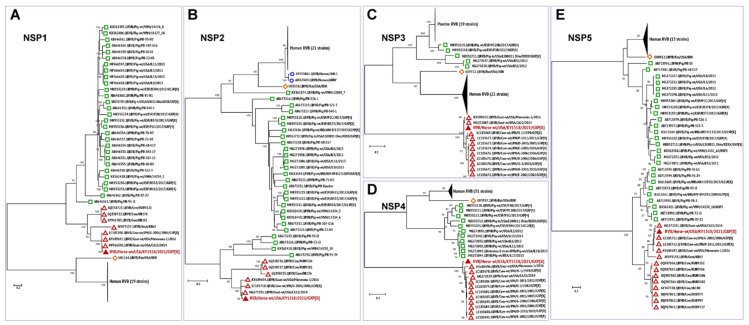
Phylogenetic trees of the complete protein-coding nucleotide sequences of the group B rotavirus VP4 (**A**), VP6 (**B**), VP7 (**C**), NSP4 (**D**), and NSP5 (**E**) genes. Maximum-likelihood analysis in combination with 1000 bootstrap replicates was used to derive trees based on the nucleotide sequences encoding respective proteins. The RVB strains are color-coded as human (blue open circle), murine (open orange diamond), porcine (green open square), and ruminant (red open triangle), with RVB/Horse-wt/USA/KY/1518/2021 (filled red triangle) highlighted in red font. A scale bar representing the number of nucleotide substitutions per site is also shown in each panel. Bootstrap values are shown above branches to the left of major nodes.

To further understand viral evolution, we performed phylogenetic analysis of the novel equine group B rotavirus, and representative group B viruses of various species acquired from the NCBI database (https://www.ncbi.nlm.nih.gov/nuccore/?term=Rotavirus+B, accessed on 20 April 2021) using MEGA X [44]. The evolutionary history of all the 11 segments of RVB/Horse-wt/USA/KY/1518/2021/GXP[X] was analyzed individually by constructing maximum-likelihood trees, using the best nucleotide substitution models suggested by the goodness-of-fit criteria in MEGA X (Figure 2, Figure 3 and Figure 4). The best substitution models inferred for the maximum-likelihood trees were the general time-reversible model with gamma distribution and invariant sites (GTR+G+I) for VP1, VP2, VP3, VP6, and NSP1 encoding genome segments; the Hasegawa-Kishino-Yano model with gamma distribution and invariant sites (HKY+G+I) for VP4 and NSP4; the Tamura 3-parameter with gamma distribution and invariant sites (T92+G+I) for VP7; and the Tamura-Nei with gamma distribution and invariant sites (TN93+G+I) for NSP2, NSP3, and NSP5 segments. For the analyses, complete or near-complete nucleotide sequences of the RVB strains of swine, human, caprine, bovine, and murine origin were included, while the gaps or missing data were removed during the analyses. For each taxon, the bootstrap value was determined from 1000 replicates to verify the tree topology. The total number (given in parentheses) of RVB sequences used for the phylogenetic analyses for each segment were VP1 (*n* = 54), VP2 (*n* = 48), VP3 (*n* = 46), VP4 (*n* = 51), VP6 (*n* = 66), VP7 (*n* = 70), NSP1 (*n* = 57), NSP2 (*n* = 58), NSP3 (*n* = 59), NSP4 (*n* = 55), and NSP5 (*n* = 54).

Phylogenetic analyses of the genes encoding all the structural and non-structural proteins revealed a high level of divergence of ruminant RVB strains from the porcine, murine, and human RVB (Figure 2, Figure 3 and Figure 4), which is similar to what has been previously described [27,45]. In general, all 11 genes of this novel equine RVB strain clustered more closely with the RVB strains of ruminant origin than with those of porcine, murine, or human strains, indicating the likelihood of a cross-species transmission event between ruminants and equines. The phylogenetic analyses also showed that the RVB/Horse-wt/USA/KY/1518/2021 shared a common ancestor with the bovine and caprine RVB strains. Moreover, for all RVB segments (Figure 2, Figure 3 and Figure 4) except VP3 (Figure 2C), NSP3 (Figure 4C), and NSP4 (Figure 4D), the novel ERVB is more closely related to the ruminant RVB strains from Japan and the USA than to the Indian bovine RVB cluster. The sequences for VP3, NSP3, and NSP4 segments of Indian bovine RVB strains were not available in the database and hence were not included in the phylogenetic analyses. Additionally, RVB/Rat/USA/IDIR clustered more closely with the human RVB strains than with the ruminant and porcine RVB strains for all 11 segments.

Phylogenetic characterization of the viral core assembly proteins VP1, VP2, and VP3 is shown in Figure 2. The VP1 gene encoding RNA-dependent RNA polymerase (RdRp) protein is the largest protein that aids transcription and genome replication [46]. The VP1 gene of the RVB/Horse-wt/USA/KY/1518/2021 clustered more closely with a caprine RVB strain RVB/Goat-WT/USA/Minnesota-1/2016 originating from the USA, followed by three bovine strains (DB176, RUBV226, and RUBV282) from India (Figure 2A). The VP2 gene encodes the most abundant structural protein that forms the outer core protein, which is essential for RNA binding and RdRp activity [47]. The gene encoding the VP2 segment of RVB/Horse-wt/USA/KY/1518/2021 shared a common ancestor with the bovine RVB strains of Indian origin and clustered more closely with two caprine strains from the USA, namely RVB/Goat-wt/USA/CA22/2014 and RVB/Goat-WT/USA/Minnesota-1/2016 (Figure 2B). The VP1 protein forms an enzyme complex with the VP3 capping enzyme, which catalyzes the addition of a 5′ cap on the viral RNA [48]. Similar to VP1, the VP3 segment of the RVB/Horse-wt/USA/KY/1518/2021 strain formed a group with RVB/Goat-WT/USA/Minnesota-1/2016, clustering with the Japanese RVB strains of bovine origin (Figure 2C).

The outer capsid proteins, VP4 and VP7, form the outermost layer of the rotavirus and possess diverse biological properties involved in receptor binding, tissue tropism, and immunogenicity [49,50,51]. These two proteins are also primary targets of virus-neutralizing antibodies. Like VP1 and VP3 genes, the VP4 segment of the RVB/Horse-wt/USA/KY/1518/2021 grouped with the caprine strain from the USA and bovine strains from India, clustering more closely with RVB/Goat-WT/USA/Minnesota-1/2016 (Figure 3A). VP6, the inner capsid protein, is the most conserved protein across rotavirus groups and is the basis of group demarcation in the new rotavirus classification system [9]. VP7 is the second most abundant protein that interacts with host cell receptors and elicits neutralizing antibody responses. While the inner capsid protein VP6 clustered more closely with the caprine strain RVB/Goat-wt/USA/CA22/2014 (Figure 3B), the outer capsid protein VP7 shared a close phylogenetic relationship with bovine RVB strains, clustering more closely with RVB/Cow-wt/USA/MN10-1/2010/G3P[X] (Figure 3C). The VP7 segment of the novel equine RVB strain was most closely associated with the bovine and caprine strains from the USA and Japan in the G4 group, followed by the Indian bovine strains that were initially designated G4 strains, but recently reclassified and placed into the G3 genotype (Figure 3C) [27,52]. In the case of VP6, RVB/Horse-wt/USA/KY/1518/2021 belonged to the I3 genotype, along with the rest of North American, Japanese, and Indian ruminant RVB strains (Figure 3B).

Similar to the structural proteins, the evolutionary history of the non-structural proteins of the RVB/Horse-wt/USA/KY/1518/2021 also revealed that all five non-structural proteins (NSP1, NSP2, NSP3, NSP4, and NSP5) clustered more closely with the RVB strains of ruminant origin than with the porcine, murine, and human RVB strains, thus supporting the proposition that RVB was transmitted from ruminants to the equine species (Figure 4). Among the ruminant RVB strains, the strains from Japan and the USA were phylogenetically closer to RVB/Horse-wt/USA/KY/1518/2021 than the Indian strains (Figure 4A,B,E). The non-structural protein, NSP1 protein, is the most variable of all the rotavirus proteins and is associated with inhibition of the host antiviral innate immune responses [53]. Here, NSP1 of the equine RVB virus shared a close phylogenetic relationship with caprine RVB strains, RVB/Goat-wt/USA/CA22/2014 and RVB/Goat-wt/USA/Minnesota-1/2016 (Figure 4A). While NSP3 promotes the translation of viral RNA by inhibiting the host translation machinery, the NSP4 protein is an enterotoxin, which acts as a viroporin and is an important virulence determinant [5]. Both NSP2 and NSP5 are involved in viroplasm formation, and NSP5 interacts with both NSP2 and core replication complex VP2–VP1–VP3, aiding the RNA encapsidation and core assembly during the early replication phase [54]. The non-structural proteins NSP2, NSP3, NSP4, and NSP5 clustered closely with the RVB/Goat-wt/USA/CA22/2014 (Figure 4B–E). Overall, the phylogenetic analyses of the genome segments revealed that all 11 segments of RVB/Horse-wt/USA/KY/1518/2021 shared a common ancestor with the bovine and caprine strains originating from the USA, Japan, and India, which agrees with our BLAST-based results (Table 1 and Table 2).

### 3.4. Detection of Equine Rotavirus Group B by RT-qPCR

A standard RT-qPCR assay targeting the VP7 gene (Assay I) was designed and used to confirm the presence of ERVB in fecal samples derived from outbreaks of foal diarrhea. The detection limit was ~34 copies and the cut-off Ct was 34. Assay I did not detect the VP7 gene product of rotavirus group A (simian RVA, SA11 strain), confirming the specificity of the assay (data not shown). The results showed that 23 (23 of 33, 69.69%) samples from diarrheic foals, derived from a total of 18 equine farms, were strongly positive for ERVB, with cycle threshold (Ct) values between 10.65 and 25.68 (Table 3). The fecal samples with higher viral loads (lower Ct values) seemed to come from foals of less than 3 days of age. In contrast, all nine fecal samples from clinically healthy foals tested negative in the RT-qPCR assay. The VP7 gene-targeting RT-qPCR detection results were further validated by a separate RT-qPCR assay based on the NSP2 gene (Assay II), which are summarized in Table 3. Both assays were in complete agreement in the detection of the ERVB genome in these clinical samples qualitatively as well as quantitatively.

## 4. Discussion

Rotavirus groups A and B are significant enteric pathogens that cause diarrhea of variable severity in humans and domestic animals [12,16,23]. The horse is a unique species in which only group A rotaviruses are frequently found in diarrhea outbreaks worldwide, especially in foals aged 60–90 days [32,42,55]. To date, only one previous study found a single horse testing positive for group B rotavirus (2.7% RVB-positive rate) [43]. Both ERVA G3P(12) and G14P(12) strains have been isolated from samples of affected foals, with some foals co-infected with both strains [30,37,38]. In this study, we present the first evidence that the rotavirus group B of ruminant-origin has emerged in horses, and this novel virus can play an etiological role in outbreaks of diarrhea in neonatal foals, as demonstrated here. Linking rotavirus group B to this highly contagious series of outbreaks of foal diarrhea is also supported by the poor responses of the affected foals to antibiotic treatment, as well as seemingly ineffective vaccinated mare-derived ERVA-specific maternal antibodies in affected foals against this new virus. In this study, ERVB was detected in nearly 70% of foal diarrhea cases. Despite a small sample size, the prevalence of ERVB in diseased foals was similar to that reported in ERVA-associated foal diarrhea outbreaks [26,55,56,57], highlighting a critical need for further investigation due to the important impact of foal diarrhea on the equine industry.

RVB is genetically and antigenically distinct from RVA. In addition to horses, other established host species for RVB include humans, rats, swine, cattle, lambs, adult sheep, kids, and adult goats [16,27,58,59,60,61]. Among RBV of different species, human, porcine, and rat RVB lineages are more related to each other in their respective genome sequences, while RVB lineages from bovines, ovines, and caprines form a distinctive group [27,45]. Sequence analysis in this study showed that the equine RVB is more closely related to ruminant RVB than to RVB isolates from humans, pigs, and rats. Like RVA, RVB can cause sporadic or epidemic diarrhea in humans and agricultural animals. RVA is thought to be more prevalent than RVB in humans with increased disease severity in infants and higher transmissibility [3]. Nevertheless, RVB appears to be associated with diarrhea in human adults and older children over 15 years of age [16,62,63]. These clinical features are also reproduced in agricultural animal species. For example, group B rotaviruses can cause an epidemic or sporadic diarrhea in both calves and adult cows [19,60]. RVB detection rate in clinical diarrhea samples appears to increase with age in pigs [64,65]. Overall, there has been an upward trend in the frequency of RVB-associated diarrhea outbreaks in farm animals, especially piglets [59,66]. Along the same lines, two recent studies demonstrated that 49 and 71% of diarrheic piglets tested positive for RVB, respectively [59,65]. Despite lacking a recent update in small ruminants, RVB was proposed as one of the commonest causes of rotavirus diarrhea in neonatal lambs in England and Wales in the 1980s [61,67]. Widespread RVB infection in domestic animals has been further supported by serological evidence demonstrating a high incidence of RVB infection in some farm animal species (97% for pigs, 71% for bovines, and 91% for small ruminants) [68]. Equine RVB is more closely related to ruminant group B rotaviruses in terms of its genome sequence. Whether equine RVB causes a high incidence of infection and clinical diarrhea in both foals and aged horses similar to what has been observed in RVB infections in ruminants needs to be investigated.

The combination of a large number of sequencing reads and a 69.69% detection rate in clinical samples from diarrheic foals suggests that ERVB is the causative agent for this extensive series of diarrhea outbreaks in neonatal foals. This assumption is also supported by the absence of other significant enteric pathogens in clinical fecal samples, and the rapid course and highly contagious nature of the disease that has been observed, with some farms experiencing a 100% morbidity. Nonetheless, we realize that neonatal foal diarrhea is a complex, multifactorial problem that often involves an interplay among pathogens, host immunity, and environmental factors [69]. In addition, successful recovery of diarrheic foals after medical therapy has prevented us from conducting histopathological studies and characterization of viral replication in the intestinal tract. Further animal challenge experiments and pathogenesis experiments are required to demonstrate the role of ERVB in foal diarrhea and to determine the disease severity and clinical importance of infection in horses.

Numerous studies have shown that group A rotaviruses have a huge potential for cross-species transmission, and there were several reports on the emergence of equine-like or bovine/porcine-like rotaviruses in humans [70,71,72,73,74]. As such, zoonotic transmission of animal group A rotaviruses to humans has been widely appreciated in the field, and these animal RVs can cause diarrhea in humans, especially in children [21,23]. In contrast, little is known about whether animal group B rotaviruses can jump to and cause diarrhea in humans. In addition, the inter-species transmission of rotavirus B has not been demonstrated previously. The phylogenetic characterization of structural and non-structural gene segments of this novel RVB/Horse-wt/USA/KY/1518/2021 GXP[X] revealed a close association between all 11 genome segments with those of ruminant RVB strains isolated from the United States, Japan, and India. Interestingly, the equine RVB appears to be more closely related to the caprine and bovine RVB strains in the United States than to strains from the Asian countries. Specifically, VP7 and NSP4 segments of the equine virus (Figure 3 and Figure 4) grouped closely with cow strains, while its other nine segments (Figure 2, Figure 3 and Figure 4) related closely to caprine strains. It is possible that a reassortment event between cow and caprine strains may have occurred, giving rise to this ERVB, and as a result, enabling the new virus to jump species and cause enteric disease in neonatal foals. The work we have presented here suggests for the first case of mammalian-to-mammalian (ruminant-to-equine) host transfer event for RVB, highlighting the similarity between group A and B rotaviruses in terms of their evolution and potential for interspecies transmission. Further investigations are required to determine the zoonotic importance of group B rotaviruses as well as to define viral determinants that promote the emergence of ruminant RVB in horses.

The discovery of a novel group B rotavirus in diarrheic foals of the 2021 foaling season in central Kentucky provides little information about when the virus emerged in horses and whether virus infection might occur in other regions of the USA or in other countries. The widespread, high morbidity of ERVB infection in foals, as demonstrated in this study, may suggest that rotavirus B-associated diarrhea might be happening elsewhere, which warrants further investigation. It is intriguing to note that in the 1995 foaling season in Kentucky, there was a non-A rotavirus-associated diarrhea outbreak in foals reported at 24–48 h of age that resembled what was observed in this year’s outbreaks of foal diarrhea [75]. Samples were tested negative for group A rotavirus, coronavirus, and bacterial pathogens, but rotavirus-like particles were visualized in fecal samples by electron microscopy. It should be noted that the 1990s was a decade when numerous cases of group B rotaviruses were detected in farm animals, including calves and pigs [19,76]. Hence, it is possible that group B rotavirus may already have been circulating in horses around that time. This speculation seems to be further strengthened by the results of a clinical investigation of RVA, RVB, and RVC in horses in Germany involving samples collected from 1999 to 2013 in which one horse was found RT-PCR positive for rotavirus B [43]. A retrospective study on archived serum and fecal swabs would be required to track down when and where this novel virus emerged in horses. In addition, despite the evidence presented in this study pointing out that transmission on the ruminant–equine interface is a probable driver for the observed foal diarrhea outbreak in central Kentucky, natural spillover from an unknown intermediate host such as wildlife species, e.g., deer, should be considered in parallel towards establishing the origin of ERVB-associated foal diarrhea.

In summary, we identified a novel group B rotavirus that was associated with a widespread problem of diarrhea in neonatal foals. The findings reported in the present study raise several interesting questions. Does this equine RVB have the capability of generating a viable reassortant with currently circulating group B rotaviruses, especially those affecting ruminants? If so, could such a reassortment allow RVB to diverge, having a broader tropism with greater pathogenicity in foals and perhaps other animal species? What is the underlying mechanism that has driven the transfer of RVB to horses? Due to the widespread nature of rotavirus group C in cattle and swine populations, can group C rotavirus, like group B, emerge and cause diarrhea in equines? Future elucidation of these questions will provide insights into the ecology, virology, and pathobiology of equine group B rotavirus.

## Figures and Tables

**Table 1:** BLASTn analysis of the eleven genome segments of RVB/Horse-wt/USA/KY1518/2021.

Genome Segment ¦ Protein ¦ Length(nt)	Best BLASTn Hit Virus ¦ Accession Number ¦ Identity
1 ¦ VP1 ¦ 3503	RVB/Goat-wt/USA/Minnesota-1/2016 ¦ KY689687.1 ¦ 93.19%
2 ¦ VP2 ¦ 2889	RVB/Goat-wt/USA/Minnesota-1/2016 ¦ KY689688.1 ¦ 97.82%
3 ¦ VP3 ¦ 2340	RVB/Goat-wt/USA/Minnesota-1/2016 ¦ KY689689.1 ¦ 95.04%
4 ¦ VP4 ¦ 2324	RVB/Goat-wt/USA/CA22/2014 ¦ MG272136.1 ¦ 97.40%
5 ¦ NSP1 ¦ 1252	RVB/Goat-wt/USA/Minnesota-1/2016 ¦ KY689691.1 ¦ 95.75%
6 ¦ VP6 ¦ 1221	RVB/Goat-wt/USA/CA22/2014 ¦ MG272162.1 ¦ 96.34%
7 ¦ NSP3 ¦ 1007	RVB/Goat-wt/USA/Minnesota-1/2016 ¦ KY689693.1 ¦ 97.84%
8 ¦ NSP2 ¦ 961	RVB/Goat-wt/USA/CA22/2014 ¦ MG271985.1 ¦ 97.90%
9 ¦ VP7 ¦ 773	RVB/Cow-wt/USA/MN10-1/2010/G3P[x] ¦ JQ288103.1 ¦ 96.37%
10 ¦ NSP4 ¦ 697	RVB/Cow-wt/JPN/IS-1/1999/G3P[x] ¦ LC185678.1 ¦ 94.26%
11 ¦ NSP5 ¦ 764	RVB/Goat-wt/USA/CA22/2014 ¦ MG272051.1 ¦ 96.41%

**Table 2:** BLASTp analysis of the eleven putative open reading frames of RVB/Horse-wt/USA/KY1518/2021.

Genome Segment ¦ Protein ¦ ORF (aa)	Best BLASTp Hit Virus ¦ Accession Number ¦ Identity	Protein Function
1 ¦ VP1 ¦ 1158	RVB/Goat-wt/USA/Minnesota-1/2016 ¦ ASV45167.1 ¦ 97.15%	RNA-dependent RNA polymerase (RdRp)
2 ¦ VP2 ¦ 937	RVB/Goat-wt/USA/CA22/2014 ¦ AUG44960.1 ¦ 99.15%	Outer core protein; essential for RdRp activity
3 ¦ VP3 ¦ 763	RVB/Goat-wt/USA/Minnesota-1/2016 ¦ ASV45169.1 ¦ 97.38%	Catalyzes the addition of 5′ cap on vRNA
4 ¦ VP4 ¦ 759	RVB/Goat-wt/USA/Minnesota-1/2016 ¦ ASV45170.1 ¦ 97.10%	Essential for attachment to host cell
5 ¦ NSP1 ¦ 320	RVB/Goat-wt/USA/CA22/2014 ¦ AUG44808.1 ¦ 96.25%	Interferon antagonist
6 ¦ VP6 ¦ 391	RVB/Goat-wt/USA/CA22/2014 ¦ AUG45028.1 ¦ 98.98%	Essential for transcription of double layered particles
7 ¦ NSP3 ¦ 296	RVB/Goat-wt/USA/Minnesota-1/2016 ¦ ASV45174.1 ¦ 99.66%	Inhibits host mRNA translation; promotes vRNA translation
8 ¦ NSP2 ¦ 300	RVB/Goat-wt/USA/CA22/2014 ¦ AUG44851.1 ¦ 99.33%	Packaging of vRNA; viroplasm formation
9 ¦ VP7 ¦ 247	RVB/Cow/Nemuro ¦ BAA78609.1 ¦ 99.60%	Interacts with host cell receptor
10 ¦ NSP4 ¦ 208	RVB/Cow-wt/JPN/IS-2/2002/G3P[x] ¦ BAW98439.1 ¦ 96.63%	Acts as viroporin; enterotoxin
11 ¦ NSP5 ¦ 166	RVB/Goat-wt/USA/CA22/2014 ¦ AUG44917.1 ¦ 97.59%	NSP5: interacts with VP2; viroplasm Formation NSP6: interacts with NSP5

**Table 3:** Detection of ERVB in clinical fecal samples of foals by RT-PCR.

Farm	Foal	Ct (Assay I)	Ct (Assay II)	Age (Days)	Clinical Status
A	A1	20.9	21.0	3 d	Diarrhea
	A2	13.1	13.9	<5 d	Diarrhea
B	B1	24.5	28.8	2 d	Diarrhea
	B2	18	19.8	2 d	Bloody diarrhea
	B3	19.1	18.6	3 d	Diarrhea
	B4	11.6	15.1	2 d	Diarrhea
	B5	23.4	28.9	2 d	Diarrhea
C	C1	15.2	13.7	<7 d	Diarrhea
D	D1	25.6	24.0	<7 d	Diarrhea
E	E1	ND	ND	<7 d	Diarrhea
F	F1	ND	ND	<7 d	Diarrhea
G	G1	ND	ND	<7 d	Diarrhea
H	H1	15.3	16.5	3 d	Diarrhea
	H2	ND	ND	12 d	Diarrhea
I	I1	11.3	12.7	<3 d	Diarrhea
J	J1	17.3	17.4	3 d	Diarrhea
	J2	17.3	17.7	<2 d	Diarrhea
	J3	10.6	9.6	<2 d	Diarrhea
	J4	13	11.4	<3 d	Diarrhea
	J5	21	22.3	2 d	Diarrhea
K	K1	15	18.2	2 d	Diarrhea
L	L1	ND	ND	1 d	Normal
	L2	ND	ND	3 d	Normal
M	M1	ND	ND	<5 d	Normal
	M2	ND	ND	<5 d	Normal
	M3	ND	ND	<5 d	Normal
	M4	ND	ND	<5 d	Normal
	M5	ND	ND	<5 d	Normal
N	N1	13	11.9	1 d	Diarrhea
	N2	20.1	19.1	3 d	Diarrhea
O	O1	ND	ND	12 d	Normal
P	P1	ND	ND	<5 d	Normal
Q	Q1	15.5	15.3	<7 d	Diarrhea
R	R1	ND	ND	<19 d	Diarrhea
S	S1	ND	ND	4 d	Diarrhea
	S2	ND	ND	4 d	Diarrhea
	S3	ND	ND	3 d	Diarrhea
	S4	30.3	28.1	4 d	Mucoid diarrhea
T	T1	15.8	18.7	3 d	Bloody diarrhea
	T2	14.2	15.2	<2 d	Diarrhea
U	U1	ND	ND	<7 d	Diarrhea
V	V1	ND	ND	<2 d	Diarrhea

Note: ND = not detected.

## Data Availability

Data were submitted to Genbank under accession nos. MZ327688-MZ327698.
